# Estimating social contacts in mass gatherings for disease outbreak prevention and management: case of Hajj pilgrimage

**DOI:** 10.1186/s40794-022-00177-3

**Published:** 2022-09-01

**Authors:** Mohammadali Tofighi, Ali Asgary, Ghassem Tofighi, Mahdi M. Najafabadi, Julien Arino, Amine Amiche, Ashrafur Rahman, Zachary McCarthy, Nicola Luigi Bragazzi, Edward Thommes, Laurent Coudeville, Martin David Grunnill, Lydia Bourouiba, Jianhong Wu

**Affiliations:** 1 York University, 4700 Keele Street, Toronto, ON M3J1P3 Canada; 2 University of Manitoba, Manitoba, Canada; 3 Sanofi, Canada; 4 Oakland University, Rochester, USA; 5 University of Guelph, Guelph, Canada; 6 Massachusetts Institute of Technology, Cambridge, USA

**Keywords:** Social Contacts, Mass gathering, Hajj, Disease Transmission, Agent-Based Simulation, COVID-19, AnyLogic

## Abstract

**Background:**

Most mass gathering events have been suspended due to the SARS-CoV-2 pandemic. However, with vaccination rollout, whether and how to organize some of these mass gathering events arises as part of the pandemic recovery discussions, and this calls for decision support tools. The Hajj, one of the world's largest religious gatherings, was substantively scaled down in 2020 and 2021 and it is still unclear how it will take place in 2022 and subsequent years. Simulating disease transmission dynamics during the Hajj season under different conditions can provide some insights for better decision-making. Most disease risk assessment models require data on the number and nature of possible close contacts between individuals.

**Methods:**

We sought to use integrated agent-based modeling and discrete events simulation techniques to capture risky contacts among the pilgrims and assess different scenarios in one of the Hajj major sites, namely Masjid-Al-Haram.

**Results:**

The simulation results showed that a plethora of risky contacts may occur during the rituals. Also, as the total number of pilgrims increases at each site, the number of risky contacts increases, and physical distancing measures may be challenging to maintain beyond a certain number of pilgrims in the site.

**Conclusions:**

This study presented a simulation tool that can be relevant for the risk assessment of a variety of (respiratory) infectious diseases, in addition to COVID-19 in the Hajj season. This tool can be expanded to include other contributing elements of disease transmission to quantify the risk of the mass gathering events.

## Introduction

Mass gathering events are a major risk factor for the spread of the severe acute respiratory syndrome coronavirus (SARS-CoV-2), which causes the COVID-19 pandemic. The role of mass gathering events in the global outbreak of the disease has been proved in several countries [1]. According to the World Health Organization, mass gatherings can be defined as “events attended by a sufficient number of people to strain the planning and response resources of a community, state or nation” [2]. The Hajj (pilgrimage), the yearly pilgrimage to the holiest city of Mecca, Kingdom of Saudi Arabia, and one of the five pillars of the Islamic creed represents the largest annual mass gathering worldwide. During the Hajj event, pilgrims perform rituals and prayers in mass in some predefined indoor and outdoor spaces, often in very crowded conditions sometimes up to six persons per square meter [3]; under these conditions, respiratory infectious diseases could easily affect a large percent of pilgrims [4].

Although disease transmission during the Hajj has been a concern for decades, it has become one of the key issues in recent years, particularly in 2009 during H1N1 influenza, in 2012 MERS outbreaks [3, 5], and recently in the COVID-19 pandemic. Although the Hajj event has rarely been canceled in the past, major historical disease outbreaks such as the plague of 1967 have caused the cancellation of the pilgrimage. Recent disease outbreaks have been treated through the implementation of specific public health interventions such as mandatory vaccination (i.e. polio and meningitis), education campaigns, and country-specific restrictions, such as the restrictions applied to the countries impacted by the Ebola crisis [5]. Given that a large number of pilgrims take part in the Hajj from more than 180 countries [6, 7], it can potentially serve as a superspreading event, and an outbreak can have significant global impacts.

With the emergence of the COVID-19 pandemic, concerns were raised about mass gathering events including the Hajj. The Hajj for 2020 was not fully canceled. Instead, Saudi Arabia's government decided to organize a very small pilgrimage with the total number of pilgrims limited to only 1,000 [8]. Participants were randomly selected from nationals of 160 countries who were already residing in Saudi Arabia [4]. This allowed a much easier and more practical physical distancing among the pilgrims in all major Hajj sites, otherwise, the risk of virus transmission would have been very high [9, 10]. For example, Yezli & Khan (2020) estimated that the total number of primary COVID-19 cases per one million participants to be around 1,392 cases, including 472 imported cases [10]. This could go up to 4,872 after adjusting for secondary cases, which could easily overwhelm the hospital and ICU capacities in the cities hosting Hajj-related programs. In 2020, age limitation, general health condition, negative COVID -19 tests, and medical examinations of pilgrims, in addition to screening, specific quarantining, maintaining a physical distance of 1.5 meters from each other, wearing face masks, following hygiene protocols, and close communications with the public health staff were the preventive measures in the Hajj event [4]. Moreover, all local service providers were subject to regular COVID-19 screening and testing [11]. In 2021, Saudi Arabia's government increased the number of annual Hajj pilgrimage to 60,000 pilgrims. In addition to previous measures such as age limitation [12, 13], vaccination against COVID-19 was mandatory for the pilgrims [14]. Implementing these health and safety measures, resulted in no cases of COVID-19 or other illnesses among pilgrims during the 2020 and 2021 events.

The emergence of more transmissible new variants and the uncertainty that still exists about the effectiveness of vaccines on different COVID-19 variants and the duration of the protection afforded by the various vaccines has further complicated the situation for the mass gathering events including 2022 Hajj planning. Several factors such as crowding, the health of the attendees, and the type and location of meetings contribute to the transmission of diseases among participants in mass gathering events [15]. Before COVID-19, the most common outbreaks at the mass gatherings involved vaccine-preventable diseases, mainly measles and influenza, but also mumps and hepatitis [16]. However, it was found that the transmission of various communicable diseases such as gastrointestinal infections that may not be prevented by vaccination have been recorded in association with mass gatherings [16]. Therefore, evaluation, mitigation, and communication of the risk of mass gatherings play a crucial role in the decision-making process of holding events. According to WHO's technical guidance on COVID-19 [17], specific features of the event such as crowd density, type or purpose of the event, duration, and count, age, profession, mode of travel of participants as well as nature of contact between participants should be considered in the risk assessment for mass gatherings during the COVID-19 pandemic [18]. Current evidence suggests that the virus transmits mainly between people who are in close proximity to one another, often within 1 metre, and increasing the count and duration of contacts increase the risk of transmission in conjunction with mass gathering events [17].

Measurement of contacts between participants in mass gathering events is a time and cost-consuming process and to the best of the authors' knowledge, there is no such measurement has been presented for the Hajj ritual. In order to better inform our understanding of the potential contact dynamics during the Hajj event, we have developed a simulation tool that estimates the potential number and duration of risky contacts in one of the Hajj main sites, the Grand Mosque, where almost every pilgrim visits several times during the pilgrimage. The information obtained regarding risky contacts can be used to assess the feasibility of physical distancing measures as well as to estimate the risk of disease transmission under different policy choices and scenarios. In the next section, the details of modeling are described. These details can be used as a guide for the simulation of motions and contacts in other mass gatherings events. Section 3 presents the results of simulations and section 4 discusses the results and concludes the paper.

## Materials and Methods

### Hajj rituals

**Fig 1:**
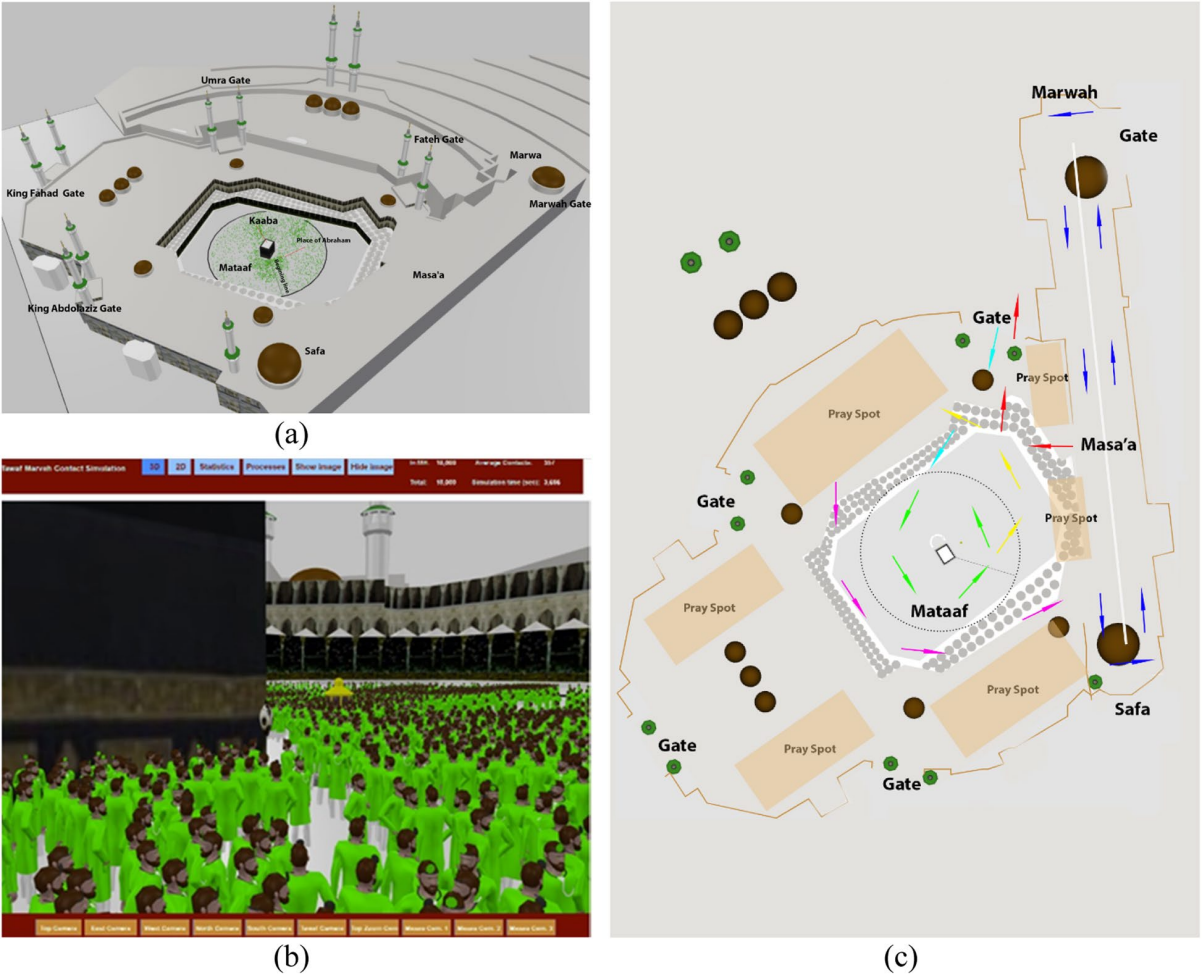
Masjid-Al-Haram **a** and **b** 3D Model, **c** Pilgrim’s path in the ritual that is begun from one of the gates and ended at the same or another gate. Arrows show one of the paths in the order of cyan, green, yellow, purple, blue and red (Refer to Video 1 and Video 2)

Pilgrims usually arrive in Mecca and perform some voluntary rituals in preparation for the mandatory rituals in Masjid-Al-Haram, called *Tawaf* and *Sa’ay*. Tawaf is performed in Mata’af, an open-air space, and around the Kaaba and Sa’ay is performed in the Masa’a building, an enclosed space (Fig 1a and b). On the first day of the obligatory ritual, all pilgrims travel to Mina and stay a day in Al-Mashaer inside tents or in open spaces. In the evening, they travel to Muzdalifah and stay there overnight in open spaces (no tents). Pilgrims' travel from Masjid-Al-Haram to Mina, Al-Mashaer, and Muzdalifah are often done through buses, train, minivans, taxis, and cars. Pilgrims stay in Mina for four days. They stay in camps while performing their rituals during the day. There are some trips between Mina and Masjid-Al-Haram during this period to perform Tawaf and Sa’ay. On the last day of their obligatory rituals, pilgrims go back to Masjid-Al-Haram from Mina and perform the last Tawaf and Sa’ay before leaving.

In this study, we focus on rituals that are performed repeatedly in the Masjid-Al-Haram complex (Tawaf in Mata’af and Sa’ay in Masa’a Building). In this study we focused on the Hajj rituals that are performed in Masjid-Al-Haram because this place is the most frequently visited place during the hajj pilgrims for obligatory and optional rituals as well as daily prayers. The complex has three floors while rituals can be performed on all floors, most pilgrims prefer to perform them on the first floor. To limit the scope and computational time, we only consider and simulate the first floor. To perform the Tawaf ritual, the pilgrims circle around Kaaba seven times counterclockwise. After completion of Tawaf, prayer is performed in designated prayer areas around Mata’af. The prayer on the first-floor area is covered but has an open connection to the uncovered Mata’af area. The prayer is followed by the Sa’ay ritual during which pilgrims run or walk seven times between Safa and Marwah points in Masa’a. The maximum Mata’af radius is about 75 m but the Tawaf is often performed in areas near the Kaaba. Tawaf begins from the corner of the Kaaba with the Black Stone and ends up with an average total distance of 1.5 km after the completion of the seven rounds. The open area around the Kaaba on the ground floor is about 15,000 m^2^. The Mata’af and Ottoman constructions around Kaaba can accommodate up to 72,000 people in a praying position [19]. The crowd density in Tawaf during the peak of the Hajj season and also during the month of Ramadan used to reach up to 8 people per square meter [20]. This density varies from 6 to 7 people per square meter near the Kaaba to 1 person per square meter in a distance of about 40 meters from Kaabah [21]. Previous studies and crowd models have shown that the upper limit throughput of the Mata’af area for efficient and safe Tawaf is about 30,000 Tawafs per hour [20]. The distance between the Safa and Marwa is around 450 m, therefore, seven trips back and forth sum up to roughly 3.6 km. This area is divided into two 16 m wide corridors. Recently, the Masjid-Al-Haram has a much higher throughput due to the increased capacity of the Masa’a building [20]. Measurements indicate that the current maximum rate of entrance and exit from the Masjid-Al-Haram can reach up to 55,000 to 65,000 pilgrims per hour in peak seasons [20]. Pilgrim speed in the Mata’af area is a function of the pilgrims' densities, time of the day, and age of pilgrims. There are fluctuations in the speed due to turbulence in the pilgrim flux, and oscillation on the pilgrims' paths caused by shock waves which are affected by the repulsive forces between the pedestrians in the high-density crowd [21]. The average measured speed varies from 0.3 m/s in the higher-density areas to 1.1 m/s in lower-density areas [21].

### Simulation method

In this study, we used a mix of agent-based modeling and discrete events simulation techniques. Agent-based models can be used to model the pedestrians' behaviors in crowds [19]. To simulate the motion of pilgrims in Masjid-Al-Haram, we developed a multiscale simulation model in AnyLogic software (www.anylogic.com). AnyLogic is a multimethod simulation modeling platform that allows simulating pedestrian dynamics. Pedestrian movement in AnyLogic is modeled according to a social force model observing rules of physics. In the social forces model, the mass and dimension of each pedestrian, desired speed of the pedestrian in the absence of interactions, the direction of movement (toward attraction points), the repulsive force between pedestrians or pedestrians and obstacles such as walls or columns are formulated in the momentum equation.

The position and speed of the pedestrian are dynamically calculated over time [19]. Pedestrians in AnyLogic take the shortest route, avoid colliding with other objects and pedestrians by analyzing the current environment, and decide on their next steps at each time step in the model simulation. We created a 3-D form of the geometry of the Masjid-Al-Haram (Fig 1 a and b) for this model. The Kaaba, Place of Abraham, entrance gates, divider walls in Masa’a, and many other details are included in the model. We have simulated pilgrims’ movements in the Masjid-Al-Haram from when pilgrims enter the site up to the point that they finish one complete round of Tawaf and Sa’ay and leave the area. Fig 1c exhibits the simulated paths of pilgrims in Masjid-Al-Haram.

It is assumed that pilgrims randomly enter the Masjid from five main gates at a predefined rate. They go to Mata’af (following the cyan color arrows) and are randomly distributed at a distance between 0.5 m and 50 m from the Kaaba in the nearest location to their ingress gate and start circulating around the Kaaba (Tawaf). After seven rounds of circulations (following green color arrows), they leave Mata’af and go to a randomly assigned praying spot outside the Mata’af area (following yellow color arrows). Pilgrims pray there for about five minutes before leaving for Masa’a building using a path outside the Mata’af area (following purple color arrows). Pilgrims enter the Masa’a building near the Safa hill and begin their Sa’ay ritual (following blue color arrows). After seven rounds of Sa’ay, pilgrims leave the Masjid from one of the five main gates (following red color arrows).

To simulate and direct pilgrims’ movements around Kaaba in Mata’af, we first created 36 fifty-meter radial lines around Kaaba, each divided into 100 half-meter segments. Pilgrims had to cross one of these segments when passing the radial lines. Using this high-resolution setting for the path of pilgrims for the Tawaf ritual, it was possible to control the movements of pilgrims in half meters distance ranges perpendicular to Kaaba. This allowed us to set the average radius of each circulation path, the extent of movement perpendicular to the circular motion, and the density of pilgrims in different locations before running the simulation. This also allowed us to implement various movement patterns such as wavy motion, and social distancing perpendicular to the circulation in the Tawaf which had been practiced during the 2020 and 2021 Hajj. Code walkthroughs and time measurements were used to verify the simulations.

According to [21], the average speed of pilgrims was assumed to be 1.1±0.1 (m/s) in low-density areas. Since the movement of pilgrims was controlled by the embedded social forces method in AnyLogic’s Pedestrian Library, the speed of pilgrims in high-density locations was adapted based on population density. Because of the randomness of movement, different time of entrances, different lengths of circulation on each circle and time of Tawaf, differences in approaching or departing direction of pilgrims that were entering or exiting Tawaf with who were circulating, different densities and consequently various speeds formed in Mata’af. In the model, we set the rate of entrance and path of pilgrims from the entrance gates to the Mata’af, and let pilgrims randomly uniformly were distributed over 100 circles around the Kaaba. All other movements were automatically controlled by the Pedestrian Library which simulates the motion like the real pedestrians. Initial tests of the model showed that using this setting, we can simulate different numbers of populations. With a population of more than 4,000 people, some congestion occurred behind the Place of Abraham near the Kaaba, which was similar to what had been observed in the real world.

The average time that pilgrims spent in Tawaf and Sa’ay was measured for different numbers of the total population. The average time in Tawaf was about 850 seconds (14 minutes and 10 seconds) when the number of pilgrims in tawaf was below 3,000, and around 1,600 seconds (26 minutes and 40 seconds) for 3,000-5,000 pilgrims. As the number of pilgrims increases, congestions start to occur that reduce the average speed of pilgrims further. The average time that pilgrims spent in Masa’a was about 3,100 seconds (51 minutes and 40 seconds). Although a small congestion area in the Masa’a building formed when the total number of pilgrims increased, it did not induce a meaningful change in each pilgrim’s time spent in the Masa’a, for up to 10,000 individuals. These values were consistent with the kinematic motion of pilgrims with the predefined average speed in the length of the path they should pass and the measured times in the Masjid-Al-Haram [20].

### Contact calculation

Close contacts are one of the main contributors to COVID-19 disease transmission. The CDC defines a close contact as someone who is within two meters of an infected person for at least 15 minutes within a 24-hour period [22]. We adopted this definition for contact, without specifying the infection status of individuals and considering the duration of contact, to calculate the number of such events taking place. We used two approaches: 1) A Monte Carlo sampling simulation to obtain the potential contacts between individuals in an area one moment in time, and 2) A simplified version of the simulation-based contacts matrix calculator method presented by [23, 24].

For the first method, it was assumed that different total numbers of individuals with an area of 0.15 m^2^ were randomly distributed in an area with a certain geometry in a moment. Pairwise distances between all individuals were calculated. Distances less than two meters were taken as a contact. For each number of contacts, the average number of individuals who had at least one contact, and the maximum number of contacts with one other individual were calculated. The generation of the pilgrims and the calculation of distances (New simulation run) were repeated until the simulated number of contacts remains approximately constant. The results of this simulation are estimates of contacts in an area in a time step of a dynamic movement. All calculations were done using a Python script.

In the second method, [23, 24]’s methodology was used to calculate the number of contacts. In this model, each pilgrim finds other pilgrims within a two-meter radius of himself/herself (close contact) at every time step. In short, if each close person is not the same as the people that were in close contact in the previous time step, a new contact case is added to the pilgrim's contact memory. These calculations begin when a pilgrim is at the entrance gate and stop when the pilgrim leaves the Masjid-Al-Haram. The contacts are accumulated during the ritual for each pilgrim and averaged over all pilgrims. We calculated the number of contacts in different locations of the Masjid-Al-Haram and normalized it for the total number of pilgrims. In addition, we calculated the unique contacts that each pilgrim experiences during the ritual. This was done by recording the new contacts in the memory of the pilgrim without considering the time of contact.

## Results

### Total and unique contacts

**Fig 2:**
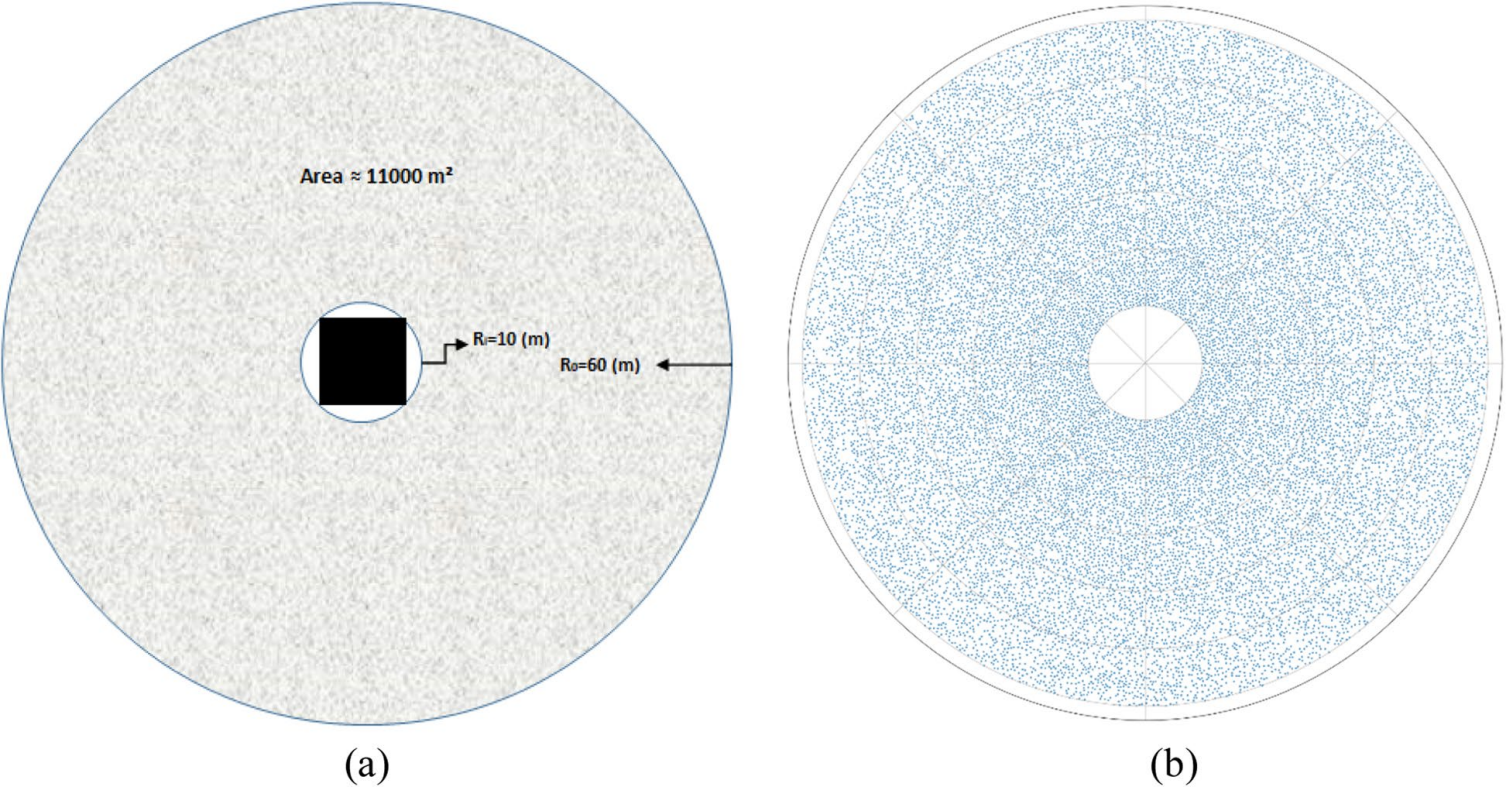
Mata’af area **a** Geometry **b** Monte Carlo simulation, random distribution of 20,000 pilgrims

Assuming the Mata’af area is a circle with a radius of 60 m, given that the Kaaba radius is 10 m, the free surface area for Tawaf would be about 11,000 m^2^ (Fig 2). The total number of pilgrims in this area can reach up to 72,000 with a density of 6-7 people per square meter (about 0.15 m^2^ per pilgrim). As a previous study indicated [20], for an efficient and physically safe Tawaf, the maximum number of pilgrims in Mata'af should be limited to 30,000 pilgrims per hour.

The rate of pilgrims' entrance to the Masjid-Al-Haram varies depending on the time of day and the season. The maximum rate in a peak season has been between 15 and 18 pilgrims per second [20]. Knowing that each efficient and safe Tawaf takes an average of 850 seconds, the maximum number of pilgrims in Mata'af should be limited to about 7,000 pilgrims that would yield an average density of 0.64 pilgrims per square meter. Therefore, the rate of the entrance to Mata'af should be restricted to remain at this level. The results of the Monte Carlo simulation for the Mata’af and the Masa’a areas are presented in Fig 2b, and Table 1. Based on this simulation, the number of contacts can exponentially increase with the increase of the total number of pilgrims. The contact parameters in the Masa’a are slightly less than the corresponding parameters in the Mata’af. Once the number of pilgrims becomes greater than 2,500 people, almost everybody experiences at least one contact. Performing the rituals is a continuous and dynamic movement and pilgrims are moving from one area to another after each step is completed. Therefore, the number of pilgrims in an area is varying in time. To be able to calculate the number of contacts for a certain number of populations, we traced and counted the number of pilgrims in Mata’af and Masa’a. In each area, when the total number of people was constant in time, i.e. in a steady-state condition, we calculated the number of contacts among the pilgrims and reported the average number of contacts per person in an hour.

**Table 1:** Number of contacts during the Tawaf and Sa’ay rituals (Monte Carlo Simulation)

	Total number of pilgrims	100	1,000	2,500	5,000	7,000	10,000
Tawaf	Total number of contacts	6.7	658	4,007	15,656	29,846	59,224
	Pilgrims with at least one contact	12	703	2,346	4,973	6,993	9,998
	Average individual contacts±0.95CI	0.12±0.03	1.30±0.03	3.21±0.03	6.20±0.06	8.51±0.04	11.85±0.03
	Maximum individual contacts±0.95CI	1.50±0.31	7.1±0.58	12.4±0.63	20.3±1.04	23.8±0.67	30.7±0.88
Sa’ay	Total number of contacts	6.4	713	4,452	17,876	35,442	72,358
	Pilgrims with at least one contact	12.5	764	2,347	4,996	6,999	9,999
	Average individual contacts±0.95CI	0.16±0.04	1.43±0.03	3.58±0.03	7.19±0.03	10.06±0.01	14.4±0.02
	Maximum individual contacts±0.95CI	1.60±0.40	6.3±0.40	11.1±0.58	16.9±0.43	22.1±0.90	27.8±0.87

To find the potential number of contacts during Tawaf and Sa’ay rituals, several scenarios were defined considering the total number of pilgrims in each of these areas while observing social distancing measures. In this study, we report the results for a total population between 1,000 and 10,000 entering Masjid-Al-Haram through the entrance gates. Table 2 summarizes the number of contacts for different numbers of the total population in the Tawaf and Sa’ay rituals, respectively.

**Table 2:** Number of contacts during the Tawaf and Sa’ay rituals (Agent-based Simulation)

Ritual	Population	Average Density (p/m^**2**^)	All Contacts (p/hr)	Unique Contacts (p/hr)
Tawaf	990	0.09	97.0	96.2
	1980	0.18	230.0	227.3
	2980	0.27	417.9	381.6
	3940	0.36	582.1	516.5
	4920	0.45	755.5	607.2
	6830	0.62	920.9	734.6
Sa’ay	998	0.07	94.2	94.0
	1995	0.14	140.0	138.4
	2992	0.21	214.4	164.9
	3989	0.28	303.4	197.1
	4980	0.35	434.7	258.2
	6960	0.48	643.6	390.5
	9920	0.69	1,259.1	556.4

**Fig 3:**
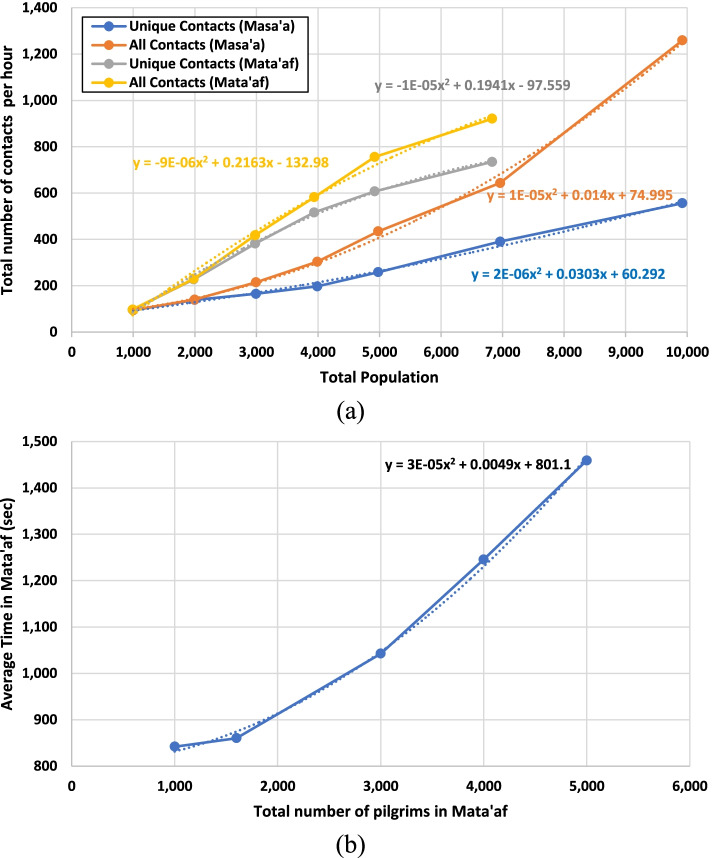
Agent-based simulation results **a** Number of contacts in the Mat’af and Masa’a areas **b** The relation between the population in the Tawaf and time spent there

Fig 3a compares the number of contacts per hour in Mata’af (Tawaf ritual) and Masa’a (Sa’ay ritual). A second-order polynomial trendline is fitted to each dataset using the least-squares method. The results show that the number of contacts increases with the number of pilgrims. Although the duration and length of the path in Masa’a are more than those of Mata'af, the number of contacts in Mata’af (in which pilgrims are mostly in rotation) is more than those of Masa’a (in which pilgrims mostly move in a straight path). This is due to the difference in the length of motion of pilgrims that are walking at different distances from Kaaba that increases the probability of becoming too close to other pilgrims. Also, more congestion occurs in the Tawaf ritual due to the fact that individuals circulating on the inner circular paths need to exit Mata’af once they finish their seven rounds, thereby meeting individuals on the outer circular paths. Fig 3b shows the average time that a pilgrim spends in the Tawaf ritual under different population sizes in the Mata’af area. As more pilgrims enter the area for this ritual, the average time spent in the area by each pilgrim increases due to the congestion and lower speed that delay the completion of the Tawaf ritual.

**Fig 4:**
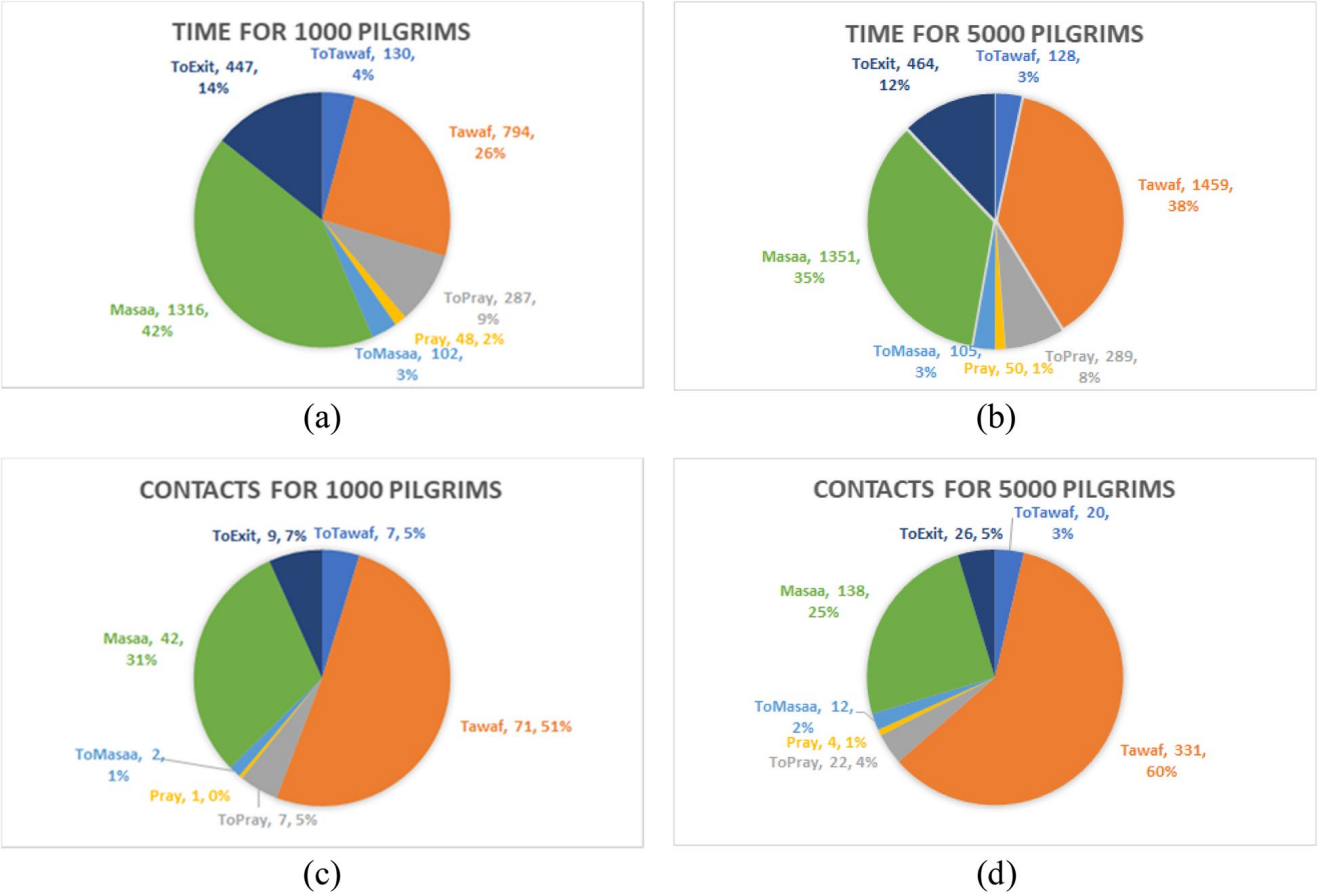
Distribution of the average number of total contacts and the average time a pilgrim spends in the Masjid-Al-Haram

Our results show that the average time spent in the different areas of the Masjid-Al-Haram does not meaningfully change as a result of changes in the total number of pilgrims, except for the Mata’af area (Fig 4a and b). The same pattern can be seen in the number of contacts distribution. More than 60% of the contacts occur when pilgrims are circulating in Mata’af (Fig 4 c and d).

### Contacts and physical distancing

**Fig 5:**
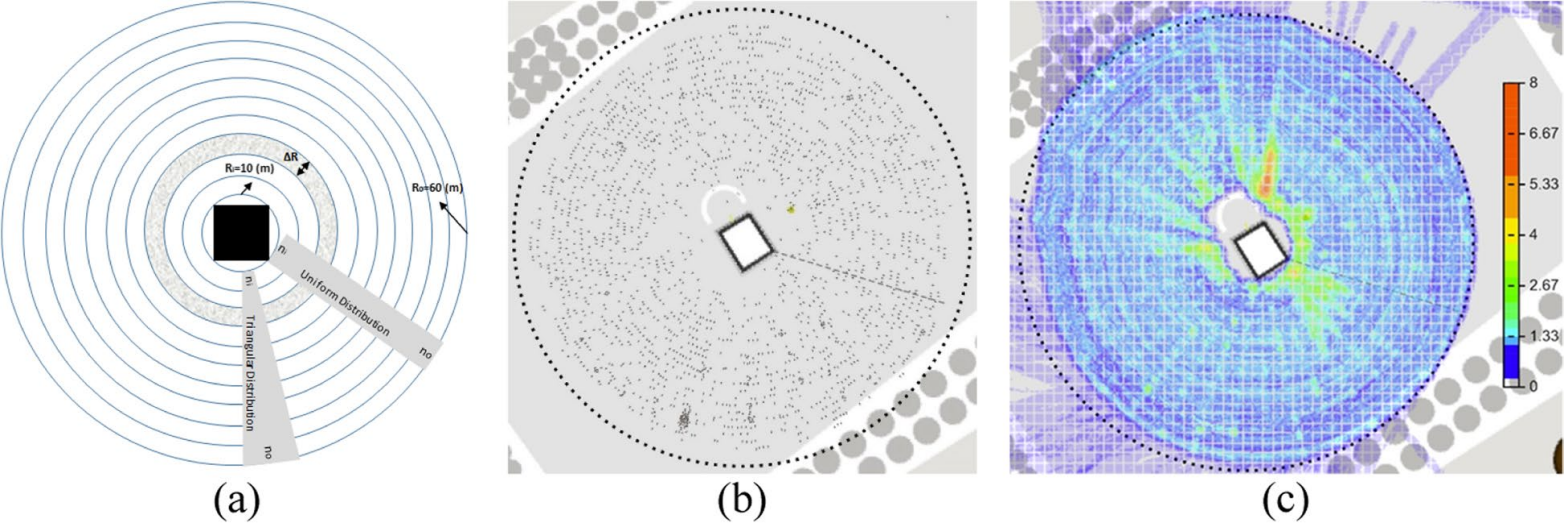
Pilgrims’ distribution in Tawaf and **a** The general setting **b** Social distancing perpendicular to the circulation (2,000 pilgrims in distance on circles with the distance of 2.0 m) **c** Density map of 2,500 pilgrims (Legend shows the number of pilgrims per square meter of area)

To examine the effect of social distancing on the number of contacts, we assumed that a controlled pattern of pilgrim distribution is applied in Mata’af during the Tawaf ritual around Kaaba. Several circular paths with a distance of 0.5 meters were assumed to be the path of Tawaf. The distribution of pilgrims entering the area was assumed to be related to the length that they should pass in the circulation. Therefore, instead of an initial uniform distribution perpendicular to Kaaba (In Fig 5a, we have *n*_*i*_ = *n*_*o*_ where *n*_*i*_ and *n*_*o*_ are the numbers of pilgrims inside and outside the circle, respectively), a triangular distribution was used (Fig 5a, *n*_*i*_ < *n*_*o*_). More pilgrims are sent to the outer circles at a larger radius. Applying this method, pilgrims are well distributed on the predefined circles (Fig 5b). However, a few local congestions are formed which are the results of the discrepancies in the speed and random direction of the circulating pilgrims (Fig 5c). This is similar to real situations where physical distancing is enforced in the Mata’af area, thus we accept those congestions. In addition, when this type of social distancing is applied in reality, the distance with the preceding pilgrim in a path should be controlled by the pilgrim and in real conditions, they cannot be forced to keep the desired distances. Therefore, we assume that the random distribution on a circular path that is controlled by the entrance rate of the pilgrims is reflective of the real situation. Using this approach, we assumed various such distances with different numbers of total pilgrims and calculated the number of contacts under each scenario. We report here the results for an average distance of 0.5 to 4 meters for 2,000, 2,500, and 3,000 pilgrims in the Mata’af area. These numbers of pilgrims correspond to densities of 0.18, 0.23, and 0.27 per square meter, respectively, which guarantees the applicability of physical distancing within the assumed parameter values.

**Fig 6:**
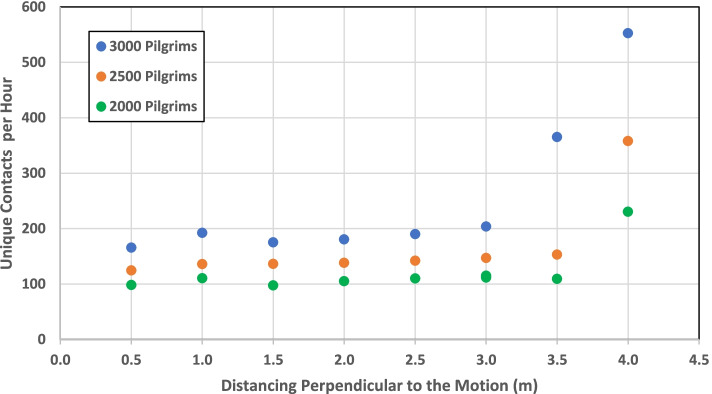
Social distancing in the Mata’af and the number of contacts

Fig 6 shows the number of unique risky contacts under different physical distancing rules among the pilgrims around in Mata’af area. With these population sizes, applying distances equal to or greater than four meters would result in more people being placed in the same length of the path, which leads to fewer front distances and more contacts. Increasing the number of contacts due to less of a gap between the pilgrims can be seen in Fig 6 for four-meter distancing.

## Discussion and Conclusion

In this paper, we presented a simulation tool that enables us to calculate social contacts among the pilgrims in Masjid-Al-Haram during the Hajj season rituals. Although simulations have been used in this area for evaluation of the risk of mass gathering, crowd management, and outbreak prevention purposes, this is the first reported study that uses simulation to estimate the potential number of contacts for public health measures, which has important implications and applications for estimating disease transmission applications and risk assessment. Implications of this social contact simulation and analysis for public health policy and regulations shall be explored in conjunction with potential social distancing measures and personal protection measures (including masking and immunization). For example, if we define the Masjid-Al-Haram basic reproduction number as the total number of transmission efficient contacts of a single infected individual at the event, then this number is given by the total number of contacts times by the transmission probability per contact. Requiring this number to be less than the unity is equivalent to the requirement that the transmission probability per contact be less than the inverse of the total number of contacts as we estimated.

Monte Carlo simulation captures a frame (a moment) of potential contacts in Masa’a and Mata’af areas while the agent-based model demonstrates the effects of dynamic motion on the contact parameters. The number of unique contacts obtained from the agent-based model is quite less than the potential contacts that might happen in the Monte Carlos simulation of Masa’a and Mata’af.

Our simulation results correspond to the real numbers reported in the literature in terms of the timing of the rituals under different population sizes. We do not have reported data for the actual number of contacts to compare and validate our findings with. However, we did not find significant inconsistencies in our results to suggest that they are unreasonable or unrealistic. Increasing the validity of this model, could come through: 1) Continue enhancing and refining the simulation to accommodate larger numbers of pilgrims; 2) Add pilgrims' usage of other floors, and 3) Develop more movement patterns and algorithms in the simulation tool.

Despite the current limitations of the model, the simulation results provide critical insights into the number of contacts and factors contributing to their occurrence that have important implications. First, our results show that as the number of pilgrims in Mata'af and Masa’a increases beyond certain levels (7,000 and more), crowd congestions start to occur. This phenomenon can sharply increase the total number of contacts. Thus, regardless of physical distancing measures taken, as soon as the number of pilgrims goes beyond a threshold, congestion is possible, and contacts will rise. Second, while close contacts are generated in all areas of Masjid-Al-Haram, our results showed that the Tawaf ritual generates the largest number of contacts due to the nature of the ritual, geometric characteristics of the area, and pilgrim movement patterns. This implies that more attention needs to be paid to this area in terms of public health measures. Third, our contact calculation algorithm is sensitive to the way close contacts are defined, as expected. We have developed an agent-based modelling platform to simulate and assess interactions among pilgrims in Masjid-Al-Haram during the Hajj season rituals. We have demonstrated the simulation tool's usage by calculating the contact rates among pilgrims according to different scenarios of physical distancing; contact rates among pilgrims according to different numbers of individuals participating in Tawaf and Sa’ay rituals and identifying the steady-state condition for the number of pilgrims residing in Mata’af according to the different number of total participating pilgrims. In this work, we adopted the definition of a contact based on the CDC’s definition of close contact for COVID-19. We used this definition as these interactions are believed to be highly relevant to the transmission of a variety of diseases spread through the respiratory route. In this light, this simulation tool may prove to be relevant for the risk assessment of a variety of respiratory infectious diseases, in addition to COVID-19. This framework can be expanded to include other contributing elements of disease transmission for COVID-19 and other respiratory diseases such as the pilgrim's age, gen and gender, the airflow patterns, the concentration of respiratory droplets, vaccine effectiveness, characteristic of the diseases, etc. to then quantify the absolute number of infections generated according to different scenarios. These features could be incorporated into the model by adding corresponding state charts that include the effects of each element. In this light, the developments in this work are a key step toward the establishment of a risk assessment platform for Hajj season rituals. Such a platform may find its usage in weighing different scenarios according to their risk for diseases spread through the respiratory route and prove to be useful in informing the decision-making made by the local authorities. The proposed method in this paper could be adapted to other mass gatherings to assess the risk of the events and to prevent the outbreak.

## Data Availability

All the data and materials that we used in this study have been acquired from the reports and literature that have been referenced in this paper and are accessible for further reference
